# α-Tocopherol and Hippocampal Neural Plasticity in Physiological and Pathological Conditions

**DOI:** 10.3390/ijms17122107

**Published:** 2016-12-15

**Authors:** Patrizia Ambrogini, Michele Betti, Claudia Galati, Michael Di Palma, Davide Lattanzi, David Savelli, Francesco Galli, Riccardo Cuppini, Andrea Minelli

**Affiliations:** 1 Department of Biomolecular Sciences, University of Urbino, 61029 Urbino, Italy; michele.betti@uniurb.it (M.B.); claudia.galati@uniurb.it (C.G.); michael.dipalma@uniurb.it (M.D.P.); davide.lattanzi@uniurb.it (D.L.); david.savelli@uniurb.it (D.S.); riccardo.cuppini@uniurb.it (R.C.); andrea.minelli@uniurb.it (A.M.); 2 Department of Pharmaceutical Sciences, University of Perugia, 06123 Perugia, Italy; francesco.galli@unipg.it

**Keywords:** vitamin E, brain, neuroplasticity, development, adult neurogenesis, seizures

## Abstract

Neuroplasticity is an “umbrella term” referring to the complex, multifaceted physiological processes that mediate the ongoing structural and functional modifications occurring, at various time- and size-scales, in the ever-changing immature and adult brain, and that represent the basis for fundamental neurocognitive behavioral functions; in addition, maladaptive neuroplasticity plays a role in the pathophysiology of neuropsychiatric dysfunctions. Experiential cues and several endogenous and exogenous factors can regulate neuroplasticity; among these, vitamin E, and in particular α-tocopherol (α-T), the isoform with highest bioactivity, exerts potent effects on many plasticity-related events in both the physiological and pathological brain. In this review, the role of vitamin E/α-T in regulating diverse aspects of neuroplasticity is analyzed and discussed, focusing on the hippocampus, a brain structure that remains highly plastic throughout the lifespan and is involved in cognitive functions. Vitamin E-mediated influences on hippocampal synaptic plasticity and related cognitive behavior, on post-natal development and adult hippocampal neurogenesis, as well as on cellular and molecular disruptions in kainate-induced temporal seizures are described. Besides underscoring the relevance of its antioxidant properties, non-antioxidant functions of vitamin E/α-T, mainly involving regulation of cell signaling molecules and their target proteins, have been highlighted to help interpret the possible mechanisms underlying the effects on neuroplasticity.

## 1. Introduction

Neural plasticity is an “umbrella term” used to describe lasting changes to the brain occurring throughout an individual’s lifespan due to an external or internal events. This concept disagrees with the previous scientific consensus that the brain develops during a critical period in early life and then remains fixed and immutable in adulthood [1]. Indeed, it is now clear that neurons in the brain are highly plastic, responding to endogenous and exogenous stimuli, such as hormone and neurotransmitter fluctuations, and behavior, thought, and emotions, thus allowing an organism to learn and adapt to its environment [2]. Therefore, neuroplasticity can be referred to as an adaptive process, but dysfunctional neural plasticity might be induced by negative events, resulting in maladaptive processes that play a role in the pathophysiology of several neuropsychiatric conditions.

The hippocampal formation, an important brain structure involved in learning and memory and in emotions, appears to be particularly affected by plastic processes throughout the lifespan of mammalians, including human. Among the most remarkable forms of neural plasticity is the ability of the hippocampus to continuously generate functional neurons during adulthood, a highly regulated process known as adult hippocampal neurogenesis, which is integral for the hippocampus functions [3,4]. Besides structural plasticity, hippocampus is able to exhibit the form of functional synaptic plasticity known as long-term potentiation (LTP) [5], which is widely believed to be one of the main neural mechanisms by which memory is stored in the brain [6,7]. Therefore, the extended restructuring and functional remodeling of the hippocampus, according to experiential stimuli and diverse endogenous and exogenous factors, may confer important adaptive plasticity. On the other hand, the perpetual capacity for structural changes might render the hippocampus particularly sensitive to perturbations that may have adverse consequences on hippocampal function. Indeed, hippocampus is a vulnerable structure impaired by events, such as stroke, head trauma and epilepsy, and it is susceptible to damage during aging and repeated stress [8]. Plastic changes triggered by intense and prolonged negative stimuli could be responsible for disease progression; this particular aspect of neural plasticity could be referred to as maladaptive [9]. In this context, the discovery of molecules capable of enhancing hippocampal plasticity and restoring dysfunctional hippocampal plasticity in pathological conditions is one of the relevant targets of neuroscience.

It has long been suspected that specific nutrients can modulate cognitive processes and emotions, affecting neuronal function and structural plasticity. Among nutritive compounds, vitamin E is one of the factors that can influence neuroplasticity, exerting effects on many different aspects of plasticity-related processes in both physiological and pathological brain.

The chain breaking (antioxidant) activity of this fat-soluble vitamin is essential to protect polyunsaturated lipids of cell membranes, lipid bodies and lipoproteins from the peroxidatic activity of free radicals, and many of its biological functions and regulatory effects in human and animal tissues have been ascribed to this effect so far (recently reviewed in [10]). However, besides antioxidant action, vitamin E exerts a wide range of non-antioxidant activities, mainly by affecting signaling pathways and gene expression [10,11,12,13]. In this review, we aimed at describing and discussing the role of vitamin E, with particular attention paid to α-tocopherol (α-T, the isoform showing the highest in vivo bioactivity and bioavailability) on neural plasticity processes occurring in the hippocampus during brain development, structural and functional synaptic remodeling, cognition, and pathology.

## 2. Vitamin E Structure and Mechanisms of Actions

Vitamin E was discovered in 1922 by Evans and Bishop [14] as a dietary factor necessary for reproduction in rats, and is a natural antioxidant [15] acting as peroxyl radical scavenger and chain breaker of lipid peroxidation [10]. Eight forms of this vitamin have been identified as relevant for human and animal nutrition that are categorized into tocopherols (T) and tocotrienols (T3), with the latter bearing an unsaturated side chain condensed in position 2 of a chroman ring. Each of these two subfamilies is further categorized as α-, β-, γ- or δ-forms, which are defined by the number and location of methyl groups on the chromanol ring. The hydroxy group in position 6 of the chroman ring is the active site involved in the H atom donation effect essential for the chain breaking (anti-peroxidatic) activity of this vitamin [16], whereas the side chain is involved in the docking of vitamers in the lipid structure of cell membranes and lipoproteins. Among the vitamin E isomers, RRR-α-T has the highest in vivo bioavailability and bioactivity tested with different protocols, and also shows the highest H-donating activity in vitro [17].

In the liver tissue, it is bound by a specific transport protein—the α-T transfer protein (α-TTP) that ensures its preferential binding (at least with a 10-fold higher affinity than in the case of the second most abundant form of vitamin E in the human organism, e.g., α-T) and transferring to nascent VLDL (very low density lipoprotein) for distribution in circulation and thus in peripheral tissues. This preferential binding appears to protect this form from excretion and catabolism as it occurs for the other forms. In this respect, the different forms are discriminated by the liver so that only α-T is retained and distributed to the cellular membranes of tissues, whereas the other forms are rapidly metabolized and excreted with the same mechanism of long-chain fatty acids and lipophilic xenobiotics [10].

As a consequence, RRR-α-T is the actual form of vitamin E that is considered essential for humans also representing the reference molecule on which the relative biopotency of the other forms is calculated [17,18]. As described elsewhere [19,20], bioactivity and levels of T and T3 forms show substantial differences in human tissue. α-T3 has been shown to have similar or even higher antioxidant activity than α-T in some in vitro models [21,22]. However, tocotrienols are present only in very low amounts in tissues since the aforementioned selectivity of liver uptake and the scarcity in the human diet strongly limit the availability of this and other forms alternative to α-T [20]. As a result, in the brain, which is very rich in highly unsaturated fatty acids, α-tocopherol represents 99.8% of the vitamin E content and no tocotrienols are detected [23]. Since the first evidence of the regulatory effect that α-T produces on protein kinase C activity, which dates back to early 1990s, an impressive series of studies have reported on the signaling function of this vitamin often suggesting the apparent independence from its antioxidant properties which still remains a matter of debate [10]. The different methylation patterns of vitamers promote selective differences in the signaling function of T and T3 isoforms [24] and this suggested a role for the chroman moiety as the signaling domain of the molecule. The control of the phosphorylative activation of some kinases appears to be a distinctive mechanism of action for the main forms of vitamin E in human tissues, which appears to depend on protein phosphatase (PP) enzyme regulation. For instance, α-Tocopherol but not β-tocopherol, activates protein phosphatase 2A, decreases protein kinase C activity and attenuates smooth muscle cell proliferation at physiological concentrations, and in a similar fashion α-T was proposed to inhibit the protein kinase C (PKC)-dependent assembly of nicotinamide adenine dinucleotide phosphate (NADPH)-oxidase complex at the plasma membrane and superoxide production stimulating PP (1 or 2A form) pathways of microglial cells and Akt-PKB dephosphorylation is prevented by α-T, but not γ-T [12], pretreatment in murine xenograft of human prostate carcinomas through the site-specific dephosphorylation of Akt, a process mediated through the pleckstrin homology (PH) domain-dependent recruitment of Akt and PHLPP1 (PH domain leucine-rich repeat protein phosphatase, isoform 1) to the plasma membrane [25]. PHLPP1 could also be involved in the α-T and γ-T phosphate dependent regulation of VEGF (vascular endothelial growth factor) expression in HEK293 cells, a response that occurs by the coordinated signaling of phosphatidylinositol-3-kinase γ (PI3Kγ) and PKB [10]. That signaling function is speculated to originate within the different lipid environments of plasmalemma and organelles, where it is delivered consequently to trafficking and subcellular distribution elements (lipid-lipid and lipid-protein interactions) that target and discriminate between the different forms of vitamin E and between this vitamin and other fat-soluble factors [26]. Some of the cytosolic and membrane-associate proteins containing hydrophobic domains that may allow the sensing, binding and subcellular trafficking/docking of vitamin E have been tentatively identified, and include for instance the human tocopherol-associated protein 1 (hTAP1/SEC14L2) [10]. Other examples of signaling proteins that respond to vitamin E in the proximity of the membrane are the complex Nrf2/Keep1 [27]. This complex stabilized in the inactive form by disulphide bridges is a redox sensitive component of the stress adaption response with transcriptional effects on detoxification and antioxidant genes that include, among others, heme oxygenase, naphtoquinone oxidoreductases, aldehyde dehydrogenases and the entire battery of genes responsible for the biosynthesis and detoxification metabolism of glutathione such as g-glutamylcysteine synthase subunits, glutathione transferase and peroxidase isoenzymes. Furthermore, vitamin E has been proposed to be a ligand of nuclear receptors such as PXR that after activation and heterodimerization with RXR translocates into the nucleus to promote the transcription of genes primarily involved in the metabolism of vitamin E and other lipids [20]. Other orphan receptors involved in the vitamin E signaling through RXR transactivation include some members of the PPAR family. The homo- or hetero- dimerization of these receptors can control different groups of genes involved in drug metabolism and cytoprotection, immuno-inflammatory response, lipid and energy metabolism, and cell cycle regulation [27]. The structural homology between vitamin E and the PPARγ agonist troglitazone has suggested a specific role for this vitamin in the gene regulation response of this PPAR isoform with roles in the control of lipid biosynthesis and inflammatory pathways of different tissues [28]. Recent evidence confirmed this effect of α-T in human hepatocarcinoma cells with a mechanism that may sustain the same cytochrome P450-dependent metabolism of vitamin E and that of long-chain fatty acids such as arachidonic acid and its eicosanoid derivatives. Again, the reported effect of vitamin E on COX-2 activity could promote the signaling and transcriptional function of PPARγ through a lowered generation of prostaglandin E2a (PGE-2γ), which is a physiological PPARγ inhibitor [29]. This role of vitamin E as a regulator of lipid signaling may involve other aspects that include the fact that it helps in preventing the nonspecific, radical-mediated, peroxidation of signalling PUFAs (polyunsaturated fatty acids) such as ARA (arachidonic acid) and DHA (docosahexaenoic acid), which are metabolized to bioactive lipid mediators via lipoxygenase enzymes such as 12- and 12/15-lipoxygenases, and even 5-lipoxygenase. This frame in the lipid signaling of vitamin E is expected to occur at the interface with the enzymatic role that cellular peroxidases have in regulating the flux of signaling lipid peroxides in the different cellular and are intimately connected with its role of chain breaker that actually produces lipid peroxides from peroxyl radical forms of PUFAs. The same PP-mediated effects of α-T on the phosphorylative activation of cellular kinases and tyrosine kinase (TK)-receptors, such as PDGF (platelet derived growth factor) receptor, could be explained with this mechanism.

### Methodological Issues Concerning Experimental Manipulations of Vitamin E Intake

In early studies, Yang and Desai [30] reported that in rats following a wide range of long lasting protocols of vitamin E intake, α-T concentration in liver and plasma roughly doubled, indicating that α-T continues to accumulate over time. Consistently, Machlin and Gabriel [31] found that, subsequent to administration of high levels of vitamin E, plasma tocopherol concentration continued to increase with time in several mammals, including rats, monkeys and humans. Besides plasma, they found that a variety of tissues (liver, adipose tissue, heart, lung, skeletal muscle, and brain) increasingly accumulate tocopherol for the duration of the supplementation. Collectively, these findings suggest that α-T saturation is not easy to reach and that not only the doses, but also the duration of supplementation protocol influence vitamin E concentrations in all tissues. Therefore, albeit physiological mechanisms controlling levels of Vitamin E in plasma and tissues are not yet fully elucidated, α-T seems to be transferred to peripheral tissues mainly via lipoprotein lipid pathways, including the uptake by lipid transfer proteins and lipases, receptor-mediated lipoprotein endocytosis (i.e., by LDL (low density lipoprotein) receptors) and selective lipid uptake that probably involves SR-BI-mediated HDL uptake [32]. As for the brain, vitamin E crosses the blood–brain barrier by means of these mechanisms, and its brain distribution is affected by the local expression of α-T transfer protein [33,34]. Brigelius-Flohè in her review on Vitamin E [35] reported that other lipid binding proteins may be involved in regulating vitamin E brain bioavailability. However, studies performed on animals and human [36,37] suggest that the replacement of endogenous α-T in the brain by supplementation with exogenous α-T is slower than in other tissues, thus indicating a low metabolic rate and turnover of this vitamin in brain cells. Nonetheless, bioavailability and metabolism of vitamin E affect brain susceptibility to lipid peroxidation [38]; indeed, vitamin E levels in rat brain dentate gyrus have been found to be inversely associated with lipid peroxide concentrations [39]; moreover, lipid peroxidation can be prevented by vitamin E supplementation in several animal models of drug-induced epilepsy [40,41]. It is worth noting, however, that vitamin E biokinetic is associated with a wealth of inter-individual variations, including dyslipidemias and/or certain genetic factors, which could account for the impaired efficacy of vitamin E supplementation [42]. As a consequence, from a translational point of view, it is necessary to keep in mind the influence of biochemical and genetic factors on vitamin E bioavailability in designing vitamin E supplementation protocols in clinical studies.

## 3. α-Tocopherol and Brain Development

Vitamin E is thought to play a major antioxidant role in developing organisms. High maternal intake of vitamin E was shown to protect offspring development in rodent experimental pregnancy models, i.e., decreasing embryo malformations in gestational diabetes [43,44,45,46,47], and reducing brain atrophy and DNA damage in ethanol-exposed mothers [48], suppressing neuronal degeneration and hemorrhagic damage in fetal brain exposed in utero to neurotoxicants, such as acrylamide [49]. Despite encouraging preclinical evidence, clinical trials found no clear association in humans between maternal vitamin E supplementation and the rate of fetal malformations, preterm birth, and neonatal or perinatal death [50,51,52,53,54]. However, the high accessibility of vitamin E, together with the great interest elicited by its antioxidant role, have expanded the spontaneous attitude to increase maternal α-T intake also among the general population [51].

Extensive vitamin E supplementation during pregnancy and lactation is expected to deliver a large amount of α-T to fetus and developing offspring. The toxicity of vitamin E is thought to be low, yet some concerns on the fetal safety of early exposure to α-T excess have been raised. Evidence in humans are controversial: prospective studies have revealed a positive association between maternal vitamin E concentration and birth weight and length [55,56]; in contrast, randomised trials have shown that vitamin E supplementation is associated with an increased rate of low birth weight in normal [53] and in pathological [57] pregnancies. Teratogenic potential of vitamin E is reportedly minor in both rodents and humans [44,53,58]; however, high maternal vitamin E intake was shown to correlate with an increased risk of congenital heart defects in offspring [59]. Moreover, concerns for the safety of massive α-T intake in pregnancy have to be extended to lactation, when α-T transfer is more efficient and more readily increased via maternal intake [60], as recently confirmed in humans [61,62,63].

To this regard, the α-T-mediated modulation of PKC activity, as documented both in vivo and in vitro in different cell types, including neurons [24,64,65,66,67], could be of particular relevance. Distinct PKC isozymes [68] are differentially expressed in neural tissues, where they function as signal transducers in a variety of brain developmental processes, such as cell proliferation and differentiation, dendritic growth, synapse formation and pruning [69,70,71,72,73,74,75]. It is worth noting that alterations of PKC isozymes’ activity and/or expression are involved in brain developmental dysfunctions associated with neurotoxicant exposure occurring during gestational and perinatal periods [49,76,77]. Based on this rationale, we tested the hypothesis that maternal supranutritional intake of α-T could affect PKC function in fetal and early postnatal brain, thereby influencing neurodevelopmental processes in the progeny [78,79,80].

### 3.1. Effects on Postnatal Development

**Figure 1:**
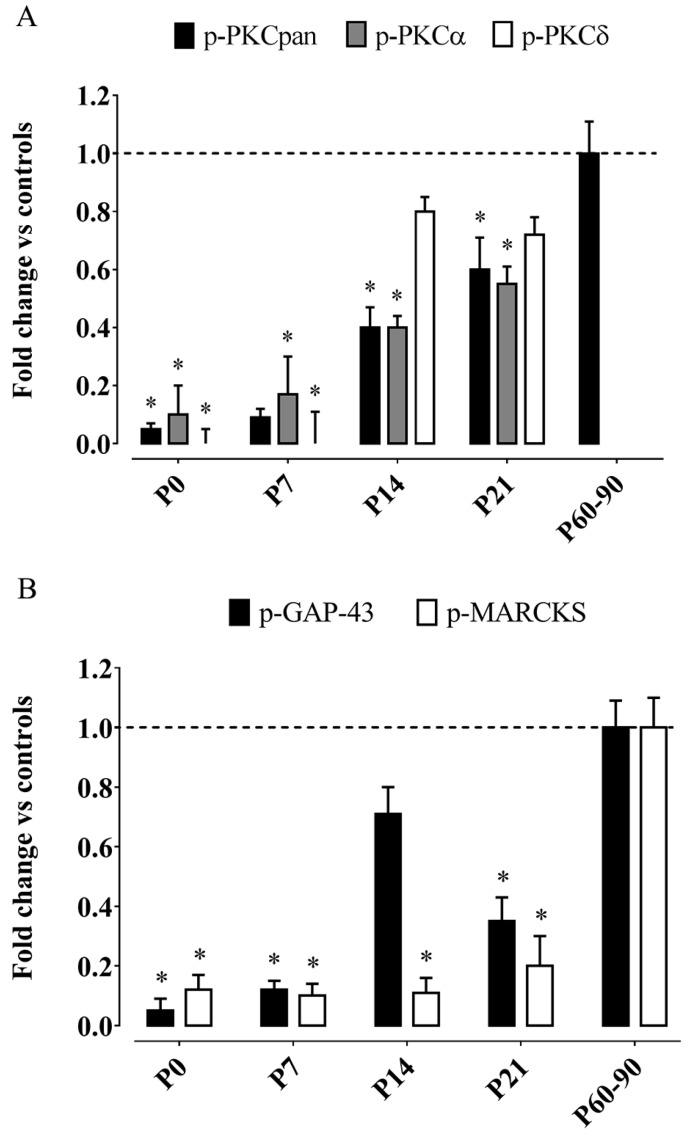
Phosphorylation of protein kinase C (PKC) and PKC substrates are reduced in developing hippocampus of α-tocopherol-exposed pups. (**A**) PKCpan, PKCα and PKCδ phosphorylation in the hippocampus of developing offspring and PKCpan phosphorylation in the hippocampus of adult offspring; (**B**) Phosphorylation of PKC substrates GAP-43 and MARCKS in the hippocampus of developing and adult offspring. Hippocampal protein extracts taken from CTRL and TREAT developing and adult offspring (at each time point, for each group, *n* = 8 animals from four different litters) were subjected to SDS/PAGE (12% polyacrylamide for PKCpan, PKCα, PKCδ and GAP-43 and 7% polyacrylamide for MARCKS) followed by Western blotting, using polyclonal phospho-specific antibody directed to PKCpan, PKCα, PKCδ, GAP-43 and MARKS. Histograms represent densitometric analyses of blots from three independent experiments (means ± S.E.M.). Representative CTRL value is shown as dashed line. Relative decreases in band absorbance values (arbitrary units) were normalized for the control band in each series. Student’s *t* test: * *p* < 0.05. Figure modified from [78]. GAP: growth associated protein; MARCKS: myristoylated alanine-rich C-kinase substrate; CTRL: control, untreated; TREAT: treated.

We fed dams a diet highly enriched in the natural RRR-α-T form, starting from two weeks before mating throughout pregnancy and lactation, providing an estimated daily intake of α-T around 1000 mg/kg/day (i.e., similar to that proposed in prevention studies). The bioavailability of maternally-administered α-T to offspring brain was assessed by HPLC (high performance liquid chromatography) analyses, revealing a two-fold increment of hippocampal α-T incorporation at birth as compared to controls, with intergroup differences remaining virtually unchanged at the end of lactation (postnatal day 21, P21). Immunoblots revealed that maternal supranutritional intake of α-T does reduce PKC phosphorylation in offsprings’ developing hippocampus, with levels of the phosphorylated, active form of PKCpan (p-PKCpan, including all kinase isozymes) being 5% of control at P0, then gradually raising to 10% at P7 and 40% at P14, to reach 60% at weaning; in adult offspring, p-PKCpan levels were similar to controls (Figure 1A). In addition, the phosphorylated forms of a conventional, Ca^2+^-dependent PKC isozyme (p-PKCα) and a novel, Ca^2+^-independent isoform (p-PKCδ) were separately analysed, showing that α-T supplementation strongly inhibits both isozymes, yet with differential timing (Figure 1A): While changes of p-PKCα followed a developmental pattern similar to p-PKCpan, p-PKCδ levels were virtually undetectable in the first postnatal week, soaring to 80% of control already at P14. Consistent with PKC inhibition, levels of phosphorylated MARCKS (myristoylated alanine-rich C-kinase substrate) and growth associated protein (GAP)-43, two PKC substrates that couple PKC signalling to plastic modifications in cell structure and motility [81,82], were found to be reduced in α-T-exposed pups (with developmental variations of MARCKS and GAP-43 activity displaying temporal profiles similar to PKCα and PKCδ, respectively; Figure 1B). PKC and its substrates are thought to play important roles in neuronal development and plasticity. PKC activity regulates neural cell proliferation, differentiation, and migration induced by many neuromodulators and growth factors [72,74], and controls activity-related circuit refinement through neuronal plasticity processes, as neuritogenesis, synaptogenesis, dendritic branching, spine formation [69,70,71,73,74,75,83,84,85]. PKC-dependent, GAP-43 phosphorylation is involved in cell growth, neurite outgrowth, synaptic remodeling and axonal guidance [81,86] and GAP-43 knock-out mice exhibit disrupted cortical maps [87]. MARCKS expression is developmentally regulated in association with neuronal migration, cortical lamination, process outgrowth, and synaptic maturation [88], while MARCKS-deficient mice show reduced brain size and abnormalities in neocortical and hippocampal lamination [89]. Since morphological, physiological, and neurochemical maturation of rodent hippocampus is virtually complete after P14 [90], developmental deviations possibly induced by PKC activity dysregulation, if any, should be fully expressed in the third postnatal week. At this time (P14), however, we have found that neuronal morpho-functional maturation in the hippocampus was virtually unaffected by maternal α-T supplementation. In fact, the morphology and the global complexity of dendritic arborization of CA1 pyramidal neurons were unchanged, as well as their basic electrophysiological characteristics (membrane passive properties and both spontaneous and evoked neuron excitability); moreover, no obvious effects were found on the expression levels (immunoblotting), as well on the topographical and laminar distribution pattern and morphological features (immunohistochemistry) of important synaptic markers, i.e., synaptophysin and spinophilin. This apparent inconsistency is not easily explained, yet some biological and methodological points are worth commenting on. First of all, we found that α-T inhibition of PKC phosphorylation is more robust in the first postnatal week, when the role of PKC-mediated signaling is thought to be minor [91]; thus, we could assume that the lack of PKC activity in early postnatal brain might be taken over by other vicarious signaling pathways. Secondly, growth-cone motility and extension can be regulated by GAP-43 also independently from its phosphorylation [92]; as we observed that maternal α-T supplementation does not influence protein expression levels of GAP-43 (but exclusively its activity, as phosphorylated form), we propose that plasticity-related functions of non-phosphorylated GAP-43 may well be kept in our model. Finally, several signaling factors other than PKC can activate MARCKS and GAP-43 as well [82,93]; considering that our antibodies specifically recognize the site phosphorylated by PKC on MARCKS and GAP-43, different PKC-independent activation of these substrates cannot be ruled out.

A key finding in our work [78] is that maternal α-T supplementation reduces the efficiency of long-term synaptic plasticity in juvenile hippocampus (P14–P21). After high-frequency stimulation, the slope of extracellular field excitatory postsynaptic potentials recorded from CA1 stratum radiatum was significantly reduced in α-T-exposed pups with respect to controls. While LTP induction was impaired, the potentiation was kept for 30 min, thus suggesting that α-T did not affect LTP maintenance. It is assumed that PKC activity is the main coordinator for processes underlying activity-dependent synaptic rearrangements [94,95]; in addition, growing evidence suggests that LTP needs F-actin cytoskeletal assembly-disassembly dynamics, thus requiring an involvement of GAP-43 and MARCKS in synaptic plasticity [96]. LTP disruption found in α-T-supplemented pups may thus be partially derived by the inhibition of PKC and PKC-substrate: Consistently with LTP impairment, indeed, we observed a vigorous decrease in phosphorylation levels of Ca^2+^-dependent PKCα isoform, whose activation was considered as a precocious event in LTP induction in Schaffer collateral-CA1 pathway [97], and of MARCKS, whose function is thought to control activity-driven plastic rearrangements in dendritic spines [98].

### 3.2. Long-Lasting Effects in Adulthood

Although underlying mechanisms are poorly understood, it was proposed that early life events producing subtle changes in brain maturation can give rise to persistent behavioral and cognitive deviations [99]. Given its long-spanned period of maturation, the hippocampus may be especially vulnerable to perinatal insults [100,101]. In our model, we have found that adult (60–90 days) rats maternally exposed to α-T loads exhibit a marked reduction of the ability to induce LTP in CA1 hippocampal slices, thus indicating that the impairment in long-term synaptic plasticity observed in juvenile offspring persists in adulthood long after suspension of tocopherol exposure, when hippocampal α-T concentration, as well as PKC and PKC-substrate phosphorylation had returned to control levels [78]. Concurrently, adult rats developmentally exposed to massive doses of α-T exhibit a different hippocampus-dependent cognitive behavior with respect to age-matched controls: in particular, performance in contextual fear conditioning (CFC) was improved, with maternally supplemented rats displaying stronger association between aversive stimulus and context, whereas spatial learning and memory abilities, as tested using Morris water maze (MWM), were impaired. Although able to acquire the task at the end of training, treated rats needed longer to develop a precise spatial preference for the goal, showing longer escape latency and path length respect to non-supplemented controls; in addition, they spent less time swimming in the goal quadrant after platform removal [79]. It is worth mentioning that spatial cognitive performance on MWM was found increased, instead of impaired, in adult offspring from dams supplemented through pregnancy and lactation with tocotrienol-rich fraction (providing 300 and 700 mg/kg of total tocopherols and tocotrienols, respectively), with better escape latency and shorter distance travelled after the third day of training, and increased memory retention at the probe test [102]. We think that such conflicting data is possibly due to different experimental study design by Nagapan et al. [102], since high doses of tocotrienols were used for supplementing dams, and supplementation was continued in offspring until they were tested, at 16 weeks postnatal.

Based on our findings, two different kinds of hippocampus-dependent learning are affected in an opposite way by the same maternal treatment, i.e., over dosages of α-T, during brain development. This observation may not be surprising per se, since differential responses to these hippocampus-dependent learning tasks were found also in adult rats following pre- and post-natal zinc supplementation, as well as in serotonin-deficient mice [103,104]; yet, an explanation is lacking. The impairment in hippocampal LTP induction could well be interpreted as a neurobiological substrate underlying the reduction in MWM performance observed in adult offspring of α-T-supplemented dams, but it does not easily fit with the improvement of CFC. Several factors need to be considered to interpreting these seemingly discrepant findings, i.e., differences in cognitive complexity inherent to the accomplishment of the two tasks, the heterogeneity of the neural circuits sub serving the two kinds of learning, and also the enhanced explorative drive found in treated rats using Y-maze and light-dark box tests (for more details, see discussion in [79]).

In sum, our data suggest that early loads of α-T, by influencing brain developmental processes, may promote permanent dysfunction in the hippocampal circuitry, thereby affecting activity-driven neuronal plasticity and hippocampus-dependent learning ability in adult progeny. The underlying mechanisms leading to such permanent effects are unclear. Certainly, the absence of gross alterations in hippocampal structure and in neuronal morpho-functional characteristics does not rule out the possibility that massive maternal intake of vitamin E may trigger more subtle alterations in offspring brain development that could play a causal role. Therefore, we decided to explore the possibility that adult offspring of α-T-supplemented dams may carry ultrastructural changes in hippocampal CA1 stratum radiatum, i.e., the very brain region where we have observed a reduction of LTP induction (and deeply involved in mediating hippocampus-related learning abilities; [105,106]). We found that the density of axo-spinous synapses (asymmetric, presumably glutamatergic; [107]) was significantly higher in maternally-supplemented adult rats as compared to age-matched controls. We believe that such aberrant gauging of synapse production/elimination balance during hippocampal maturation is mainly due to reduced synaptic pruning, since: (i) Adult PKC mutant mice display defective elimination of surplus climbing fibers onto cerebellar Purkinje cells [69,108]; (ii) At P14, we have shown that hippocampal expression and topographical distribution of synaptic markers are virtually normal [78]. Since at early postnatal ages synaptogenesis largely outpaces synapse elimination [109], an increment of synapse production rate is unlikely to occur in our model; (iii) Down-regulation of PKC and PKC-dependent, developmental-related protein phosphorylation in the hippocampus of α-T-treated rats persists into late phases of postnatal maturation [78], thus possibly affecting synapse elimination processes occurring at juvenile age. The finding that manipulations of early nutritional environment—in our case, excessive α-T maternal intake and delivery to offspring—can lead to permanent changes in neural circuit refinement is of clinical relevance, since alterations in the establishment, maintenance, or pruning of synapses have been postulated to occur in several neurodevelopmental disorders [110].

In addition, we found interesting changes in the morphological organization of neuron–glia relationships at hippocampal synapses. Adult offspring from α-T-supplemented dams showed a more extended glial coverage of presynaptic boutons at axo-spinous CA1 synapses; moreover, the percentage of synapses contacted by astrocytic endfeet at their bouton-spine junction (directly facing the site of presynaptic release) was significantly higher in treated animals, thus pointing to a surplus of hippocampal “tripartite synapses” in adult offspring of α-T-supplemented mothers. Perisynaptic astrocytes are regarded as integral synaptic elements [111,112,113]. A number of mechanisms underlying astrocytic effects on synaptic plasticity have been reported to have either facilitating or inhibiting, context-dependent roles, in several brain areas including hippocampus [114,115,116,117,118,119]. Taking into account these findings, we speculate that the enhanced astrocytic coverage of hippocampal synapses found in adult rats maternally exposed to α-T might potentially influence LTP induction mainly by increasing glutamate uptake capacity. Consistently, ceftriaxone-mediated enhancement of GLT-1 gene transcription was displayed to change GLT-1 expression and distribution, and impair activity-dependent synaptic plasticity at mossy fibre-CA3 hippocampal synapses [120]. In addition, knockout mice lacking Ephrin-A3, a molecule implicated in neuron-glia crosstalk regulating synapse morphology and function in the hippocampus [121], show impaired LTP induction and deficits in hippocampus-dependent tasks, primarily attributed to post-translational up-regulation of glutamate transporters in astrocytes and more potent glutamate elimination from the cleft [114,122].

In conclusion, we reported that maternal loads of α-T over pregnancy and lactation induce in the hippocampus of adult offspring permanent changes in axo-spinous synapse density and in neuron–glia morphological relationships at synapses, together with deficits in LTP induction and alterations in hippocampus-dependent cognitive behavior. Our findings support the general concept that stress occurring early in life, including those deriving from nutritional insults, can result in long-lasting effects in adult brain. Importantly, the increase in the doses of vitamin E used in most supplementation trials in pregnant women, ranging between 40 and 100 times the normal recommended amount [50,51,52,53], is comparable to the increase applied to the nutritional intervention of this study in the animal model. Thus, our findings underscored the need to carefully assess the safety of developmental exposure to high doses of α-T in humans.

## 4. α-Tocopherol, Synaptic Plasticity and Cognitive Functions

### 4.1. Evidence in Animal Models

During the last two decades, a large body of evidence gathered in animal models has documented that antioxidant compounds, including vitamin E, can buffer or prevent the decline in hippocampal LTP (a long-lasting increase in synaptic transmission efficacy proposed as a cellular substrate for mammalian learning and memory; [123,124]) and the impairment in hippocampus-dependent cognitive abilities occurring in various conditions associated with elevated levels of oxidative stress.

One of these conditions is aging. High levels of reactive oxygen species (ROS) and oxidative stress markers, as well as reduced enzymatic and non-enzymatic antioxidant defenses, have been found in the brain of aged animals [39,125,126,127]. Importantly, the severity of age-related cognitive deficits typically correlates with the amount of accumulated oxidative damage [128,129], thus supporting the oxidative stress hypothesis of aging (for a review, see [130]). Rescuing effects of vitamin E on age-related neuroplasticity disruption are well documented. In old rats chronically fed α-T supplemented diet, age-related increases in interleukin-1β and lipid peroxidation were reversed, and the concurrent improvement of LTP in the dentate gyrus (DG) was linearly correlated with brain α-T levels [39]. In keeping, behavioral studies have shown that aged rats supplemented with vitamin E exhibit improved learning speed and retention memory on hippocampus-dependent spatial cognitive tasks (Morris water maze) in comparison with age-matched rats fed a normal diet [131]. Moreover, supplementing rats with a combination of *N*-acetylcysteine, α-lipoic acid and α-T, was able to prevent age-dependent alterations in brain synaptosomal parameters (i.e., increased membrane potential with altered content of Na^+^ and K^+^ under both basal and stimulated conditions) and the impairment in learning and memory functions examined on T-maze [132]. Besides α-T, other members of the vitamin E family, i.e., tocotrienols (natural compounds present in select vegetable oils, as palm oil), have also been reported to rescue age-related deficits in neuroplasticity and cognition. Adult and old rats supplemented for 8 and 3 months, respectively, with a tocotrienol-rich fraction (200 mg/kg/day) displayed higher spatial learning and memory performance, reduced amount and severity of DNA damage, decreased level of oxidative markers such as malondialdehyde, as well as increased levels of antioxidant enzyme activity and brain vitamin E compared with age-matched, non-supplemented controls [133,134].

Beneficial effects of vitamin E on synaptic plasticity and hippocampus-dependent cognition have been confirmed in various pathological conditions associated with excessive ROS accumulation in the brain, as in rodent models of chronic exposure to neurotoxicant such as lead [135,136] and melamine [137], in rats fed an oxidative, high-fat high-carbohydrate diet [138], in rats exposed to sleep-deprivation [139], chronic stress [140] and traumatic brain injury [141], as well as in rodent models of diabetes [142,143] and Down syndrome [144]. When changes in redox markers were also assessed, vitamin E-induced amelioration in neural plasticity and cognitive abilities was consistently associated with a parallel reduction of brain and circulating markers of oxidative damage [135,138,139,141,143,144], thus suggesting that the radical scavenging properties of vitamin E may play an important role in rescuing neuroplasticity.

Conversely, vitamin E deprivation has been associated with a deficit in synaptic plasticity and cognitive performance. LTP in hippocampal CA1 slices was markedly reduced in rats fed for 3 months with vitamin E-deficient diet as compared to age-matched animals receiving a standard diet with about 60–65 mg of vitamin E/kg [145]. In line, young rats fed a vitamin E-deficient diet showed increased thiobarbituric acid-reactive substance (TBARS), lipid hydroperoxides, and protein carbonyls in synaptic plasma membranes, as well as hippocampus-dependent learning abilities similar to those of aged animals [146]. Moreover, reduced brain tocopherol levels in old aged, knock-out mice for scavenger receptor, class B, type I (SR-BI; critical for maintaining the homeostasis of cholesterol and α-T) were associated to LTP impairment, reduced total depolarization value (a measure of NMDA response summation when excitatory synapses are stimulated with high-frequency burst), and behavioral deficits in recognition and spatial memory task [147].

### 4.2. Possible Mechanisms

Most studies have underscored the centrality of the antioxidant functions of vitamin E in explaining its beneficial effects on neuroplasticity and cognitive behavior. In fact, redox state is thought to affect synaptic function and plasticity in a complex way: ROS are important physiological mediators of plasticity and signaling, but they can become detrimental to neuronal structure and function when they accumulate excessively in the brain, as during aging, ischemia, trauma, and neurodegenerative diseases [148]. ROS have been implicated as modulators of hippocampus-dependent learning and memory [148,149,150], and can regulate several molecules involved in synaptic transmission (neurotransmitter receptors, such as NMDA (*N*-methyl-d-aspartate) [151,152] and GABA (γ-aminobutyric acid) receptors [153]; ion channels, such as calcium, sodium and potassium channels (for a review, see [154]); SNARE (soluble NSF attachment protein receptor) proteins involved in presynaptic membrane docking [155]), as well as diverse plasticity-related signaling factors (CaMKII [156]; extracellular signal-regulated kinase, ERK [157,158]; cAMP response element binding protein, CREB [159]) in the hippocampus. Vitamin E can counteract excessive ROS-induced effects on synaptic transmission and plasticity, as documented in experimental models of oxidative stress induction. In rats exposed to hyperoxia, vitamin E was reported to prevent oxidative degeneration of lipids and proteins in nerve terminal membranes and to reverse the deficit of depolarization of the membrane surface [146]. In a similar model of hyperoxia-induced oxidative stress, vitamin E was shown to inhibit the increase of oxidative markers (thiobarbituric acid reactive substances, conjugated dienes, and protein carbonyls) in synaptic vesicles and pre-synaptic plasma membranes, and to attenuate the decrease of membrane fusion between the two plasma membranes in the nerve terminals [155], likely by preventing the oxidation and denaturation of the SNARE proteins [160,161]. Moreover, in rats injected intracerebroventricularly with proinflammatory cytokine IL-1β, vitamin E dietary manipulation was reported to block the inflammation-triggered cascade of events leading to reduced neuroplasticity, i.e., the increase in ROS production, the stimulation of c-Jun NH2-terminal kinase (JNK) and p38 activity, and the attenuation of glutamate release [162]. The vitamin E analog antioxidant Trolox prevented the reduction of GABA_A_ receptor binding capacity elicited by exposing adult rat hippocampal slices to hydrogen peroxide [153]. The notion that vitamin E-mediated rescuing of neuroplasticity is mainly attributable to its antioxidant properties gains further support from evidence showing that, in young healthy rats, thus bearing no major alterations of redox state, (i) α-T treatment does not increase the efficiency of hippocampal LTP as compared to age-matched non-treated controls [39]; and (ii) chronic vitamin E supplementation fails to improve cognitive behavior in hippocampus-dependent learning and working memory tasks ([163]; but see also [142], 2010 for data showing that supplementation with vitamin E, but only in combination with vitamin C, improves learning and memory in passive avoidance learning tasks in healthy adult rats).

Alternative mechanisms have also been proposed. A direct role of vitamin E in the modulation of LTP has been suggested by early reports showing that brief application of vitamin E was able to induce a slowly developing, long-lasting increase in excitatory post-synaptic potentials (EPSPs) of CA1 neurons in guinea pig hippocampal slices that was independent of NMDA receptor activation, while the same effect was not observed with ascorbate (thus possibly not requiring radical scavenging; [164]). Vitamin E has been shown to have non-antioxidant, relevant effects on intracellular signaling pathways, most of them mediated by the modulation of protein kinase C (PKC) activity, as reported in different tissues and cell types, including neurons [11,24,65,78]. PKC activity has been proposed as a major coordinator for processes underlying activity-dependent synaptic modifications, such as LTP induction in hippocampal CA1 and CA3 areas [94,95], and PKC-substrate molecules, i.e., GAP-43 and MARCKS, are involved in F-actin cytoskeletal assembly–disassembly dynamics observed in LTP [96]. Interestingly, a brief incubation of rat cerebrocortical synaptosomes with α-T was shown to facilitate Ca^2+^-induced glutamate exocytosis, possibly through the activation of PKC signaling and the increase of phosphorylation of MARCKS [165]. A further mechanism, seemingly not mediated by radical scavenging, has been proposed by evidence showing that vitamin E can directly affect the expression and function of post-synaptic neurotransmitter receptors involved in neurotransmission and plasticity. For example, a close interaction of vitamin E with cholinergic system in memory retention processes has been reported in studies showing that post-training intracerebroventricular administration of vitamin E affects sensitivity of cholinoceptors in the brain, potentiating the effects of receptor agonists (nicotine and pilocarpine), while attenuating those of antagonists (mecamylamine and scopolamine) [166]. In addition, chronic treatment with vitamin E was reported to increase NMDA receptor density in different brain regions, including neocortex, hippocampus and striatum, in an age-independent, likely ROS-unrelated way [167,168].

### 4.3. Human Studies

In the light of preclinical evidence, several epidemiological and longitudinal observational studies have been carried out in humans to investigate the possible association between habitual dietary intake of antioxidants and cognitive functions. Recent systematic reviews of population-based cohort studies have found moderate evidence for a protective effect of antioxidant nutrients, including vitamin E, against cognitive decline in older people [169,170]. A slower rate of global cognitive decline over 3 years was found in persons in the highest quartile of intake of the most common three antioxidants, i.e., vitamins C, E, and carotenes [169]; moreover, dietary intakes of the same nutrients have been proposed to lower the risk of Alzheimer’s disease (AD) and Mild Cognitive Impairment (MCI), with vitamin E exhibiting the most pronounced protective effects [170]. However, a systematic review based on eight cross-sectional and 13 longitudinal studies failed to confirm the beneficial role of habitual intakes of dietary antioxidants in reducing the risk for cognitive decline as well as for dementia and AD [171]. Large heterogeneity in study design, differential control of confounders (for instance, people who eat diets rich in antioxidants tend to lead healthier lifestyles, and, therefore, the effects of antioxidants in such individuals may be potentiated by other factors such as lower caloric intake and/or physical exercise), insufficient measures of cognitive performance, and difficulties associated with dietary assessment may explain these discrepancies. More recently, some evidence, though weak, for a protective effect of antioxidant dietary intake (vitamin C, vitamin E, β-carotene, lutein, flavonoids and lignans) on decline in global cognitive function, memory, processing speed, and cognitive flexibility came from the population-based Doetinchem Cohort Study [172]: regression analyses showed that lignans displayed a strong linear association with slower cognitive decline, and that people falling into the lowest quintile of vitamin E intake showed a decline in memory that was twice as fast as in all higher quintiles of intake. Moreover, data obtained from 140 non-cognitively impaired elderly subjects derived from the Cardiovascular Risk Factors, Aging, and Dementia (CAIDE) study, and followed-up for 8 years to detect cognitive impairment (mild cognitive impairment, MCI) or AD, confirmed that elevated serum levels of tocopherol (especially γ-T) and tocotrienol forms of vitamin E are associated with reduced risk of cognitive impairment, with the association being modulated by concurrent cholesterol concentration [173]. In line, low plasma tocotrienols levels have been found associated with increased odds of MCI and AD [174].

Although observational studies have brought moderate evidence that antioxidant nutrients may slow age-associated cognitive decline, data from randomized clinical trials investigating the effects of vitamin E supplementation are less encouraging. A Cochrane Database systematic review published in 2012, considering all unconfounded, double-blind, randomized trials in which treatment with vitamin E at any dose was compared with placebo for patients with AD and MCI, found no convincing evidence for benefits in prevention or progression of dementia [175], and concluded that future trials assessing vitamin E treatment should not be restricted to α-T. In line with this indication, recent neuropathological, post-mortem studies showed that γ-T concentrations were associated with lower amyloid load and neurofibrillary tangle severity, whereas high α-T was associated with lower amyloid levels only when γ-T levels were high, thus concluding that randomized trials should consider the contribution of γ-T [176]. More recently, two systematic reviews of randomized controlled trials investigating the effect of nutritional interventions on cognitive performance in older non-demented adults found no convincing evidence for clinically-relevant effects of vitamin E (either as a monotherapy or in combination with other antioxidant vitamins such as vitamin C or β-carotene) on delaying cognitive decline or the onset of dementia [177,178]. Moreover, a multi-site, randomized, double-blind controlled clinical trial recruiting adults with Down syndrome older than 50 years, with participants assigned to receive 1000 IU of vitamin E orally twice daily for 3 years or identical placebo, failed to show any effects of vitamin E in slowing the progression of cognitive deterioration [179]. Therefore, in the light of evidence coming from intervention studies, whether dietary antioxidants may constitute appropriate prevention or therapy for cognitive decline and dementia in older people remains open for debate.

## 5. α-Tocopherol and Adult Hippocampal Neurogenesis

Adult neurogenesis is a remarkable form of lifelong neural plasticity that has attracted increasing interest during the last decades: It consists of a multi-step process, resulting in generation of new neurons, which in turn contribute to neural plasticity, and have great potential for repairing the diseased or aged brain [180,181,182].

This phenomenon was introduced by Altman’s pioneering studies [183], declaring the end of the neurobiology dogma about the fixity of the central nervous system, once completed the development. Nowadays, it is recognized that new neurons are continually generated, from neural stem or progenitor cells, in discrete regions of mammalian brain [184,185], even though knowledge in humans is still limited. Hippocampus, in particular the dentate gyrus subregion, represents one of the brain areas in which neurogenesis occurs throughout the lifespan [185]. Here, new neurons show specific electrophysiological properties in the first few weeks after their birth, during which they are hyperexcitable in an otherwise mainly inhibitory environment, substantially impacting the neural network [186,187,188]. Despite the considerable progress made on the knowledge of this phenomenon, a basic question remains open, concerning the function of adult neurogenesis in hippocampal activities. However, a wealth of studies have implicated adult hippocampal neurogenesis in several brain functions, including learning and memory processes [189,190,191,192,193,194,195,196]. Moreover, recent evidence has suggested that it also plays an important role in the aetiology of anxiety disorders, depression and age-related deficits [197,198,199] and it has started to emerge as an integrator of cognition and emotion [200,201].

The great interest in adult hippocampal neurogenesis has induced several research groups to investigate the mechanisms underlying this process and regulating it, to find out endogenous and exogenous factors useful to in situ improve adult neurogenesis, as well as to allow the survival of transplanted neuronal progenitor cells. Thus, due to the tremendous work done, a wide range of factors are currently known to affect hippocampal neurogenesis. In this view, much evidence demonstrates that environmental factors and experiences, inducing neuronal activity, regulate each step of the adult neurogenesis process, from neural progenitor proliferation to new neuron maturation, synaptic integration, and survival [3,196,202]. In addition, various intrinsic factors (such as developmental morphogens, neurotrophic factors, neurotransmitters, and steroids) have been reported to influence hippocampal neurogenesis events [194,196,203,204,205]. Finally, a number of exogenous factors has been discovered to modulate hippocampal new neuron generation, among which, we have well documented the involvement of vitamin E, mainly α-T [64,67,206,207,208].

In the 90s, we found that vitamin E deficiency, induced by a diet lacking of this compound, allowed neurogenetic potential expression in rat dorsal root ganglia [209,210,211], bringing forward in time the increase in the number of primary sensory neurons normally occurring later in control animals [212]. In the same decade, on the basis of these findings, we decided to investigate whether vitamin E could play a role in neurogenesis regulation also in the adult central nervous system. To validate this hypothesis, we quantified the number of newborn cells in hippocampal dentate gyrus in vitamin E-deficient rats, by using 5-bromo-2′-deoxyuridine (BrdU) cell proliferation marker, administered over two months after the ascertainment of the decreased tocopherol plasmatic level. The results showed that adult hippocampal neurogenesis was enhanced in vitamin E deficient rats [207], suggesting that this compound could play an antiproliferative role in vivo, as it was already known to do in vitro [12,213]. However, since the actual number of BrdU-labelled cells in the dentate gyrus could be regarded as a balance between precursor proliferation and labeled cell survival, the increase in the number of BrdU-labeled cells observed in vitamin E deficiency could be due, in principle, to changes in cell proliferation, in newborn cell survival or in both. Therefore, since the protocol employed, labeling proliferating cells over a long period of time, did not allow us to choose between these hypotheses, in a subsequent work [206] neural precursor proliferation and newborn cell fate were discriminated, by counting BrdU-labelled cells in the dentate gyrus at different time points after BrdU injection (i.e., 1 and 30 days after the last BrdU injection being suitable times to study proliferation and survival, respectively) in control rats and vitamin E-deficient rats, also evaluating the occurrence of cell death by TUNEL (terminal deoxynucleotidyl transferase dUTP nick end labeling) technique. The data showed that vitamin E deficiency enhanced neural precursor proliferation, but also increased newborn cell death. In light of these results, we could explain the finding obtained in the previous work [207], assuming that vitamin E-deficiency induced an enhancement in the rate of cell proliferation that overcame cell death, allowing proliferated cells to accumulate.

Altogether, these findings indicated that vitamin E could be an exogenous factor regulating adult hippocampal neurogenesis, and boosted our interest in further demonstrating it. Hence, we decided to intraperitoneally supplement rats with low doses of the main isoform of vitamin E, α-tocopherol, to evaluate cell proliferation and newborn cell survival 1 day and 30 days after BrdU injection, respectively [64,208]. Despite the low dosage used, supplementation protocol was found to be efficacious in significantly increasing α-T tissue levels within two weeks. In keeping with the previous findings, we found that: (i) The number of newborn cells decreased after α-T supplementation, confirming the hypothesis that it is able to depress cell proliferation in vivo; (ii) More newborn cells survived in α-T-treated rats, supporting a neuroprotective role of α-T. To further validate the latter hypothesis, we studied cell death in hippocampus dentate gyrus in vitamin E-deficient, α-tocopherol-supplemented and control rats at different ages, using TUNEL technique, also characterizing the phenotype of dying-cells [67]. In this work, we quantified a higher number of TUNEL-positive cells in vitamin E-deficient rats and a lower number in α-T-supplemented rats, with respect to age-matched controls, supporting the neuroprotective effect exerted by vitamin E.

### Possible Mechanisms

Mechanisms underlying vitamin E/α-T actions on cell proliferation and newborn cell survival in adult hippocampus are not yet fully elucidated. Several papers, by a number of research groups (e.g., [11,12,13,213]), emphasize that, beside the best known antioxidant effect, this compound is able to exert non-antioxidant actions (see above Vitamin E structure and functions), modulating signal transduction and gene expression. In particular, several enzymes involved in signal transduction might be directly or indirectly influenced by vitamin E [214,215,216,217,218]; and vitamin E actions on signal transduction might translate at the cellular level in the modulation of specific gene expression [214,216,217]. Evidence showing that vitamin E is able to modulate enzymes involved in signal transduction derives from studies on protein kinase C (PKC) [219,220], which is inhibited by α-T isoform [221]. This finding has opened the way to the view that PKC could be inhibited by vitamin E-mediated non-antioxidant mechanisms. Indeed, PKC inhibition by α-T was found to occur by changing its phosphorylation state via stimulation of the protein phosphatase 2A (PP2A), through a mechanism not yet clear, and by prevention of enzyme translocation to the plasma membrane [222,223]. At the cellular level, PKC inhibition by α-T results in reduction of cell proliferation, as shown in several different cell types [219,224,225,226,227,228,229,230,231,232]. Taking into account all these considerations, we have hypothesized that mechanisms underlying α-T effects on neural progenitor proliferation could involve activation of protein phosphatase 2A, which, in turn, may cause dephosphorylation of PKC-α, thus inhibiting it [12,233]; the latter may be able to induce the phosphorylation of AP1 (activator protein 1) transcription factor, changing gene expression [234].

However, it cannot be forgotten that vitamin E is an outstanding radical scavenger within the lipophilic environment of bilayer [235] and that it has been shown that oxidative insults induce apoptosis [236]. Indeed, α-T was proposed to significantly reduce molecular events underlying apoptosis mediated by oxidized low-density lipoproteins, among which there is caspase activation [237]. Nevertheless, α-T may modulate the signal transduction pathway centered on phosphatidylinositol 3-kinase, leading to increased cell survival via induction of the anti-apoptotic Bcl-2 protein [238] and a decrease in proapoptotic Bax protein levels [239]. These mechanisms may account for the enhanced newborn cell survival we have found in adult hippocampus dentate gyrus under α-T supplementation conditions.

Moreover, it is worth noting that adult neurogenesis appears to be regulated by a novel class of modulators named microRNAs (miRNAs), short non-coding RNA that regulates gene expression at the post-transcriptional level, involved in neural stem cell proliferation and neuronal differentiation, such as miR-9, miR-124, miR-137, and miR-125 [240]. Considering that vitamin E was found to affect expression of some miR [241,242], we may speculate that vitamin E could induce epigenetic regulation of hippocampal adult neurogenesis by influencing miR expression.

In sum, our findings demonstrate that vitamin E, mainly α-T, can be considered as an exogenous factor affecting different steps of the neurogenetic process in adult rat hippocampus dentate gyrus, possibly through signal transduction modulation, resulting in gene expression alterations.

## 6. α-Tocopherol and Epilepsy

Epilepsy is one of the most common serious neurological diseases, accounting for 1% of the global burden of illness [243]. Indeed, at least 50 million people worldwide suffer from this disorder and about 100 million people experienced seizure (usually defined as an abnormal pattern of neuronal activity which includes hypersynchronization and high frequency firing of neurons) once during their lifetime [244]. Temporal lobe epilepsy (TLE) is one of the most common types of epilepsies in humans and it can negatively impact life quality, resulting in neurobiological, cognitive, psychological and social consequences. In recent years, numerous antiepileptic drugs (AEDs) have been developed, but several unmet clinical needs still remain, including resistance to AEDs found in about 30% of patients, adverse effects elicited by AEDs that can further reduce quality of life, and the lack of treatments that can prevent development of epilepsy in patients at risk. This latter issue has considerable importance, if we consider that “seizures beget seizures”, implying a role of epileptic activity in initiating the epileptogenic process but also in maintaining it, advancing epilepsy toward a more severe and chronic state. In fact, following an initial epileptic event, there is a an interval of variable duration currently referred as the latent period, during whereby several changes occur in brain structures, that are associated with the alteration of network excitability and synchronization and may account for epileptogenesis [245], even though recently it has been suggested that epileptogenesis may involve continuing changes in the neural network extending into the chronic epilepsy period [246]. Hippocampus represents a brain area affected by mesial TLE [247] and in which several structural changes occur following an epileptic event, modifying its excitability, such as cell loss and synaptic reorganization of the surviving neurons, axonal sprouting within excitatory pathway, aberrant neurogenesis [248], as well as glial and microglial activation inducing neuroinflammation which can modulate receptor function and expression [249,250,251]. All these changes could be included within the term maladaptive neural plasticity occurring in response to injury that can be responsible for the development of epilepsy. In this scenario, treatments able to reduce seizure-triggered maladaptive neural plasticity underlying epileptogenic processes have great relevance to prevent development of chronic epilepsy.

Since several studies have shown an increase in oxidative stress in epilepsy and have stated that free radicals can act as a pathogen in the disease [252,253], natural compounds with antioxidant properties were considered in preventing seizure-induced pathology [254,255]: results demonstrated that epilepsy could be partially prevented by treatment with antioxidants including SOD (Superoxide dismutase) mimetics, melatonin, vitamin C, and coenzyme Q10 [244]. Vitamin E (as α-T) was also proved to have beneficial effects in epilepsy, i.e., attenuating convulsive behavior and brain oxidative stress [40,41]. Indeed, as shown in diverse experimental models, pretreatment with vitamin E and dietary antioxidants, such as curcumin, resveratrol and ginsenosides, reduces seizure-induced oxygen and nitrogen free radicals generation on a time scale of minutes-to-hours [41,256,257,258], thereby decreasing the severity of seizures and their detrimental effects. Moreover, the use of α-T was found to reduce seizures in the Kainate (KA) model and in a model of Sudden Unexpected Death in Epilepsy, the K_V_1.1^−/−^ (K_V_1.1, potassium voltage-gated channel subfamily A member 1) model [259]. Finally, patients showing resistance to AEDs benefit from α-T treatment, improving seizure control [260,261]. In all these studies, the effects of α-T were interpreted in the light of its antioxidant role. However, as described above, neuroinflammation is also involved in the pathophysiology of epilepsy, since neuroglial activation and cytokine production exacerbate seizure-induced neurotoxicity, contributing to epileptogenesis [262,263]. In fact, seizure activity builds up the induction of inflammatory mediators, such as interleukin-1 β (IL-1β) and tumor necrosis factor-α (TNF-α) in astrocytes and microglia, followed by a cascade of downstream inflammatory events [264,265,266,267], which result in the alteration of neuronal excitability and increasing probability of seizure generation [268,269]. In this context, vitamin E has been shown to down-regulate astrocytic and microglial reactivity and glia-mediated inflammation both in vitro and in vivo in neurological diseases [270,271,272,273], suggesting antioxidant independent mechanisms.

Keeping in mind these considerations, we did a first work in which glial activation, occurring after kainate (KA)-induced status epilepticus (SE), was investigated in the rat forebrain pretreated with high dietary doses of α-T for two weeks before insult administration [78]. Expression of astrocytic and microglial antigens (glial fibrillary acidic protein (GFAP) and major histocompatibility complex II (MHC II), respectively), as well as pro-inflammatory cytokines (IL-1β and TNF-α) production were assessed. Finally, neurodegeneration was also investigated and oxidative stress as well. The effects mediated by α-T were evaluated four days after seizures, because this time point corresponds to the beginning of a transition phase (spanning between 3–7 days after SE) in which, although in absence of EEG (electroencephalogram) and behavioral seizures, specific neuroinflammatory responses are still active as an underlying event in the onset of chronic epilepsy [264,274].

We obviously considered only rats supplemented or not, which underwent a full SE based on Racine scale [275], to rule out the influence of seizure severity. We demonstrated that, four days after KA-induced SE, short-term dietary α-T supplementation was able to: (i) Promote a three-fold increase α-T brain levels; (ii) Strongly reduce brain lipid peroxidation; (iii) Significantly decrease the expression of neuroinflammatory markers, providing the first evidence of a reduction of neuroglial activation and cytokine production due to dietary α-T. In addition, besides suppressing seizure-induced neuroglial activation, α-T markedly reduced neuronal cell death occurring after SE.

**Figure 2:**
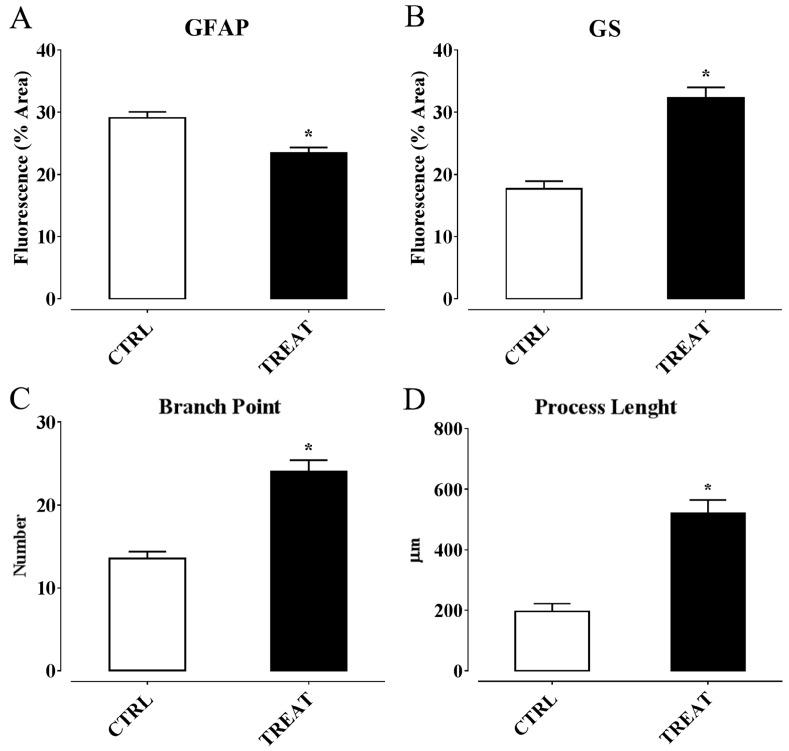

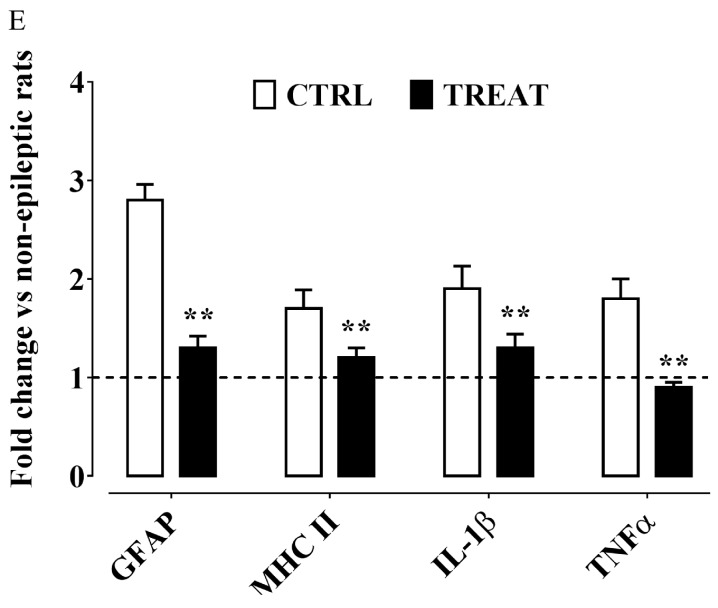
α-Tocopherol treatment decreases hippocampal neuroinflammation in kainate-induced status epilepticus. (**A**) GFAP (glial fibrillary acidic protein) immunoreactivity expressed as percent of area fluorescence; (**B**) GS (glutamine synthetase) immunoreactivity expressed as percent of area fluorescence; (**C**) Branch point number of Iba1 (ionized calcium-binding adapter molecule 1)-positive microglial cells; (**D**) Process length of Iba1-positive microglial cells; (**E**) Western blotting analysis of neuroinflammatory markers GFAP, MHC II, IL-1β and TNF-α in hippocampus protein extracts obtained from CTRL and TREAT rats. Representative non-epileptic and untreated animals’ value is shown as dashed line. Statistical analyses performed by one-way ANOVA and Tukey’s *post hoc* test: * *p* < 0.05; ** *p* < 0.01. CTRL: kainate-exposed untreated rats. TREAT: kainate-exposed α-tocopherol treated rats. Figure modified from [276]. MHC II: major histocompatibility complex II; IL-1β, interleukin 1 β; TNF-α: tumor necrosis factor α.

**Figure 3:**
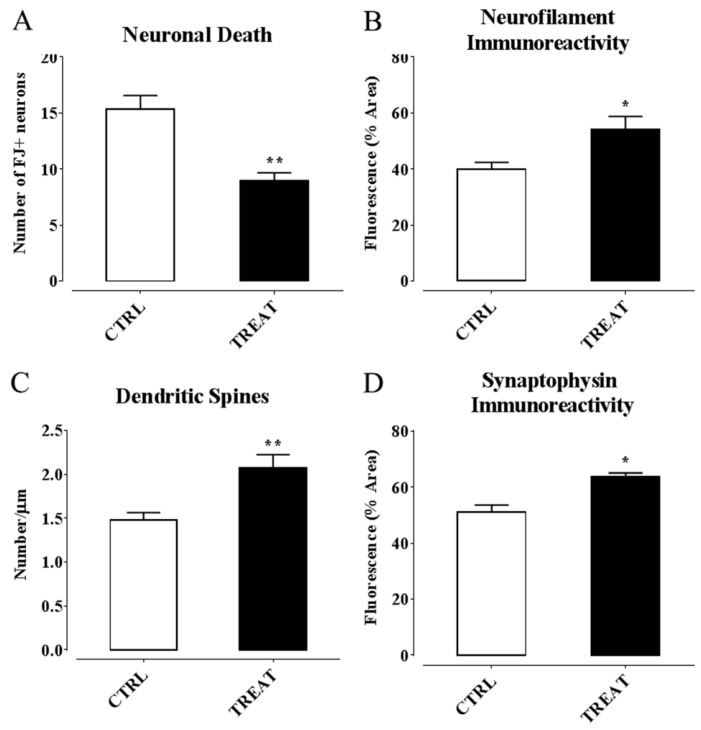
Effect of α-tocopherol treatment on hippocampal neurodegeneration induced by status epilepticus. (**A**) Degenerating neurons quantified as FluorJade-positive cells along rostro-caudal extension of CA1 field; (**B**) Neurofilament immunoreactivity expressed as percent of area fluorescence; (**C**) Dendritic spine number quantified per unit length of dendrite; (**D**) Synaptophysin immunoreactivity expressed as percent of area fluorescence. Statistical analyses performed by (**A**) Student’s *t* test: ** *p* < 0.01 and (**B**–**D**) one-way ANOVA and Tukey’s *post hoc* test: * *p* < 0.05; ** *p* < 0.01. Figure modified from [276].

Therefore, insights about an involvement of α-T in controlling epilepsy by regulating neuroinflammation processes were gaining. However, these findings derived form a study in which supplementation was performed before SE induction by α-T dietary load. Thus, we wondered if, considering these premises, a post-ictal treatment with high doses of α-T, starting early after the overt SE, were likewise effective in reducing neuroinflammation and neurodegeneration, decreasing the risk factors for epileptogenesis. Hence, we designed a second work in which α-T treatment was administered, by injection, early after SE induction, and the same time point of the previous study was considered to analyze glial and microglia activation and cytokine production, as well as neurodegeneration [276]. The main findings we obtained from this study highlighted that a brief period (four days) of treatment with α-T, initiated early after SE, induced a strong reduction of neuroglial activation and neuronal degeneration occurring following SE. In detail, we found (Figure 2 and Figure 3): (i) A decreased astrocytosis (with GFAP reduction and higher expression of Glutamine Synthase, an enzyme that contributes to preserve GABAergic inhibitory circuit activity [277]; and microglia activation (shown by a Iba1 and MHC II decrease); (ii) A downregulation of the expression of pro-inflammatory cytokines IL-1β and TNF-α; (iii) A decreased number of FluoroJade-positive degenerating neurons; (iv) An increased number of dendritic spines in Alexa-fluor-injected proximal dendrites of pyramidal neurons, according to the role of α-T in promoting synaptogenesis [65]; (v) An enhanced immunoreactivity for dendritic neurofilaments and for synaptophysin-positive axon terminals. Ultimately, our findings demonstrated that post-seizure administration of α-T was able to quench the neuroinflammatory and neurodegenerative processes of SE.

### Possible Mechanisms

**Figure 4:**
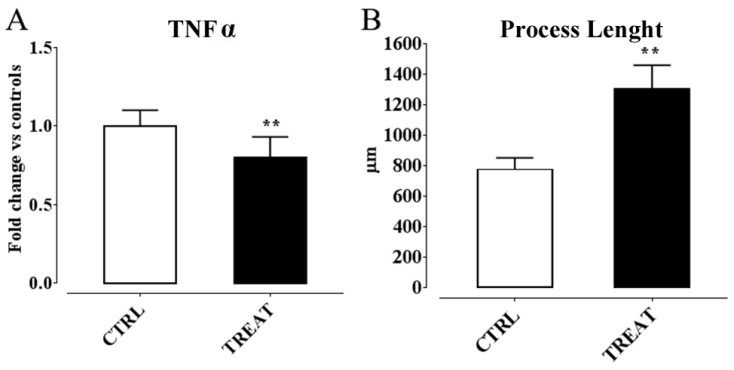
α-Tocopherol treatment per se affects neuroinflammation in non-epileptic rats. (**A**) TNF-α western blotting quantification in hippocampus protein extracts of CTRL and TREAT rats: TNF-α was significantly reduced following α-tocopherol treatment in the absence of seizures; (**B**) Process length of Iba1-positive microglial cells: The graph shows a significantly longer microglial processes, indicating the influence of α-tocopherol in determining microglia morphology and activity in normal rats. Statistical analyses performed by one-way ANOVA and Tukey’s *post hoc* test: ** *p* < 0.01. TREAT: non epileptic α-tocopherol-treated rats. CTRL: non epileptic rats’ untreated rats. Figure modified from [276].

In principle, antioxidant properties of α-T could mediate anti-inflammatory and neuroprotective effects early in the clinical management of epilepsy. Indeed, α-T brain concentration in supplemented rats drastically decreased following kainate-induced SE, together with a reduction in brain lipid peroxidation, thus suggesting that a large fraction of α-T has been actually consumed for free-radical quenching. However, some effects exerted by α-T, on neuroglial and neuronal markers affected through SE, were also detected in rats that did not undergo kainate-induced seizures (see Figure 4), thus indicating that α-T can influence structural and functional aspects of microglial and astrocytic cells, as well as of neurons, even in the absence of abnormal oxidative stress. It is increasingly emerging that diverse isoforms of vitamin E sharing similar antioxidant activity exhibit different propensity to interact with binding proteins and receptors/sensors within the cell, which ultimately regulate signaling pathways and genes related with several responses in the brain, including inflammation and cytotoxicity [278]. Moreover, α-T was found to down-regulate expression of microRNA involved in inflammation [241] as well as in astrogliosis and glial cell proliferation [242]. These findings induced to speculation that α-T could prevent the detrimental effects of epilepsy and glial activation by regulating inflammatory genes and signaling pathways with mechanisms that could be partially or completely independent from the classical antioxidant function of this vitamin [35,278].

In sum, vitamin E, mainly α-T, appears as an efficient molecule in reducing several risk factors accounting for developing of chronic epilepsy and associated cognitive dysfunction, possibly acting through its non-antioxidant properties. Taking into account that the epilepsy model system of these studies simulates the human temporal lobe epilepsy, a form difficult to treat, these findings support the potential of a timely intervention with α-T in clinical management of SE for neuroprotection and as adjuvant therapy to conventional anti-epileptic drugs that may help to reduce their dosage and adverse effects.

## 7. Conclusions

Overall, evidence presented and discussed in this review underscores the relevant role of vitamin E, mainly α-tocopherol (the isoform with the highest bioactivity and bioavailability), in modulating diverse aspects of neuroplasticity processes in mammalian hippocampus, thereby influencing hippocampus-dependent cognitive and emotional functions. Ontogeny and development, neurogenesis and neuronal differentiation, synaptic remodeling and circuit refinement are documented to be modified by vitamin E/α-T supplementation or deficiency. In addition, vitamin E/α-T is able to counteract maladaptive neuroplasticity processes, such as those induced by intense and/or prolonged insult, playing a major role in the pathophysiology of several neuropsychiatric conditions. Mechanisms implicated in vitamin E/α-T-mediated effects on neural plasticity are still a matter of debate: Besides the well-known anti-oxidant functions, these may involve alternative properties of tocopherol, such as its ability to affect neuronal cell signaling and the related gene expression.
